# Optimizing Abbreviated Breast MRI for Surveillance in Women with Personal History of Breast Cancer

**DOI:** 10.3390/diagnostics16081138

**Published:** 2026-04-10

**Authors:** Han Song Mun, Sung Hun Kim, Bong Joo Kang, Ga Eun Park

**Affiliations:** Department of Radiology, College of Medicine, Seoul Saint Mary’s Hospital, The Catholic University of Korea, Seoul 06591, Republic of Korea; im_hsm@catholic.ac.kr (H.S.M.); rad-ksh@catholic.ac.kr (S.H.K.); lionmain@catholic.ac.kr (B.J.K.)

**Keywords:** abbreviated breast MRI, breast cancer, surveillance for personal history of breast cancer

## Abstract

**Background/Objectives:** Breast MRI surveillance for women with a personal history of breast cancer (PHBC) is often limited by costs and acquisition times. This study aims to identify the optimal abbreviated breast MR (ABMR) protocol for this population by assessing the diagnostic performance of different sequence additions. **Methods:** This retrospective study included 1002 women with PHBC who underwent postoperative breast MRI with ultrafast sequences. Propensity score matching using 12 variables yielded recurrence (*n* = 21) and nonrecurrence (*n* = 42) groups with balanced characteristics. Four ABMR protocols were simulated by sequentially combining sequences: Step 1 (FAST protocol) included precontrast T1-weighted imaging (T1WI), early-phase T1WI, and subtracted maximal intensity projection (MIP). Step 2 added ultrafast MIP; Step 3 incorporated delayed-phase T1WI; and Step 4 included T2WI and diffusion weighted imaging (DWI). Three expert breast radiologists independently reviewed MRIs. Sensitivity, specificity, accuracy, and area under the curve (AUC) were assessed. **Results:** Sensitivity, specificity, and accuracy for ABMR protocols ranged from 76.2% to 90.5%, 88.1% to 92.9%, and 85.7% to 90.5%, respectively. The FAST protocol alone provided reliable performance (sensitivity: 81%; specificity: 88.1–90.5%; accuracy: 85.7–87.3%). Additional sequences yielded modest improvements, but no statistically significant differences were observed across all 3 readers (*p* > 0.05). ABMR protocols demonstrated equivalent diagnostic performance for PHBC surveillance. **Conclusions:** The FAST protocol alone provided reliable results, indicating its potential as a primary ABMR protocol. While additional sequences slightly improved specificity, they did not significantly enhance diagnostic accuracy.

## 1. Introduction

Women with a personal history of breast cancer (PHBC) face a substantial risk of recurrence. Early detection of subsequent breast cancer in the asymptomatic phase improves survival by 27–47% compared to symptomatic presentation [1,2,3]. In response to this critical need for early detection, breast MRI is widely accepted for surveillance due to its high sensitivity, particularly for mammographically occult and biologically aggressive cancers [4,5,6,7,8]. The American College of Radiology suggests that annual MRI screenings may be considered for women with PHBC, especially for those with dense breast tissue or those diagnosed before the age of 50 [9]. Despite its diagnostic advantages, routine MRI use for surveillance encounters challenges, including high costs, lengthy acquisition time, and considerable interpretation workloads [10]. Abbreviated breast MR (ABMR) addresses these barriers by significantly reducing scan and interpretation times while maintaining diagnostic accuracy comparable to that of full-protocol MRI [11]. Studies report ABMR sensitivities of 70–100% and specificities of 93–98%, with comparable or slightly higher specificity than full-protocol MRI [12,13]. ABMR also outperforms mammography and ultrasound in detecting small or early-stage recurrences [14,15,16,17,18,19]. Despite these promising findings, ABMR, as described by Kuhl [20], encompasses various protocol designs, resulting in substantial variability across studies. This variability has hindered consensus on the optimal sequence combinations. To our knowledge, no study has assessed the best ABMR protocol for women with PHBC. Therefore, this study evaluated ABMR variations in a PHBC cohort to determine the optimal surveillance protocol.

## 2. Materials and Methods

### 2.1. Study Population

This retrospective study was approved by the Institutional Review Board of our institution, which waived the requirement for written informed consent. Between June and October 2022, we identified 1037 consecutive women with PHBC who underwent breast MRI with ultrafast MIP at our tertiary referral university hospital. At our institution, annual breast MRI is recommended for postoperative PHBC surveillance, with partial reimbursement by the Korea National Health Insurance for up to 5 years post-diagnosis. This reimbursement supports MRI integration into surveillance for most patients, except those with claustrophobia or relative contraindications to contrast agents. Surveillance typically occurs every 6 months for the first 2 years and annually thereafter, using combinations such as mammography and ultrasound, mammography and MRI, or all three modalities. Clinicians select modalities based on cancer stage and patient preference.

From this cohort, exclusions included patients with insufficient clinical data or without pathological confirmation after surgery or biopsy (*n* = 21), a history of malignant phyllodes tumor (*n* = 6), and those with fewer than 12 months of follow-up after surveillance MRI (*n* = 12). The final study cohort comprised 1002 patients with 1002 MRI exams analyzed.

Pathology findings served as the reference standard for cases undergoing biopsy or excision. For malignant biopsy cases, histopathological outcomes and biopsy guidance modality were reviewed. For benign biopsy cases or those without biopsy due to negative or benign imaging findings, the absence of malignancy was confirmed through follow-up mammography, ultrasound, or MRI conducted at least 12 months after surveillance MRI. If an MRI detected a suspicious lesion without pathological confirmation, lesion stability or regression was assessed via follow-up MRI performed after at least 12 months. Recurrence was defined as malignancy confirmed in breast MRI-assessable regions, including the ipsilateral or contralateral breast, axilla, or internal mammary lymph nodes. These recurrences were initially classified as suspicious lesions based on integrated findings from all imaging modalities, including mammography, ultrasound, and MRI, as well as comparisons with prior imaging, and were subsequently confirmed as malignant by pathology. Of 1002 exams, 97.7% (979/1002) were negative, while loco-regional recurrence (ipsilateral breast/chest wall or regional lymph nodes, including axillary and internal mammary areas) or contralateral breast recurrence was detected in 23 patients.

To optimize the ABMR protocol, this study addressed the disparity between negative exams and those detecting recurrence by generating a 1:2 matched cohort using propensity score matching. The recurrence group (*n* = 21) and non-recurrence group (*n* = 42) were matched based on 12 patient and tumor characteristics from medical records: age at breast cancer diagnosis, interval between diagnosis and surveillance MRI, pathologic tumor and lymph node stage, estrogen and progesterone receptor status, human epidermal growth factor receptor 2 (HER2) status, type of surgery (breast-conserving surgery or mastectomy), and receipt of adjuvant radiation therapy, chemotherapy, and endocrine therapy.

### 2.2. MRI Techniques

Postoperative full-protocol MRIs were performed on a 3T Vida scanner (Siemens Healthcare, Forchheim, Germany) with patients in the prone position using a dedicated breast coil. The full protocol MRI included conventional DCE-MRI, acquired with a 3D T1-weighted fast low-angle-shot (FLASH) sequence. This sequence comprised one unenhanced and 5 contrast-enhanced phases, captured at 93, 176, 259, 342, and 425 s post-gadolinium DTPA injection (0.1 mmol/kg Gadovist; Bayer Schering Pharma, Berlin, Germany), followed by a 20 mL saline flush. Imaging parameters included TR/TE of 4.7/2.3 ms, a matrix of 448 × 358, a 10° flip angle, a 350 × 350 mm field of view (FOV), and a 1.0 mm section thickness with no gap in axial orientation. Ultrafast DCE-MRI was acquired using a work-in-progress prototype based on a 3D fat-suppressed VIBE sequence. This technique used incoherent k-space sampling with compressed sensing (CS) reconstruction [21], capturing 20 phases continuously during the initial contrast enhancement phase, starting simultaneously with contrast injection. Imaging parameters included a TR/TE of 3.1/1.2 ms, a spatial resolution of 0.5 × 0.5 × 1.0 mm, a 352 × 352 matrix, a 12° flip angle, a 340 × 340 mm FOV, a 1.0 mm section thickness, and a CS acceleration factor of 23. Temporal resolution was 4.2 s per phase, with a total scan time of 84 s in axial orientation. The protocol also included axial T2WI using a turbo spin-echo sequence with a TR/TE of 5000/96 ms, a 120° flip angle, 50 slices, a 320 mm FOV, a 448 × 314 matrix, and a 3 mm slice thickness, with an acquisition duration of 3 min and 23 s. Lastly, axial diffusion-weighted imaging (DWI) was performed using readout-segmented long variable echo trains with b-values of 0 and 1000 s/mm^2^. Imaging parameters included a TR/TE of 4720/60 ms, a 350 × 210 mm^2^ FOV, a 256 × 154 matrix, a 3 mm slice thickness, and a 3 min 29 s acquisition time using 9 readout segments. Apparent diffusion coefficient (ADC) maps were automatically generated via dedicated software, completing the imaging protocol.

### 2.3. Combination of Sequence and Image Analysis

**Figure 1:**
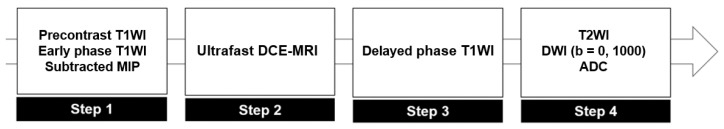
Sequential steps of the abbreviated breast MR (ABMR) protocols. Step 1 includes precontrast T1-weighted imaging (T1WI), early-phase T1WI, and subtracted maximum intensity projection (MIP), implementing the FAST protocol. Step 2 introduces MIP from ultrafast DCE-MRI without extending acquisition time. Step 3 incorporates delayed-phase T1WI. Step 4 comprises T2-weighted imaging (T2WI) and diffusion-weighted imaging (DWI) with b-values of 0 and 1000 s/mm^2^, and apparent diffusion coefficient (ADC) measurements.

Three expert radiologists with 24, 14, and 10 years of breast imaging experience retrospectively and independently reviewed MRI examinations while blinded to prior MRI results, other imaging modalities such as mammography or ultrasound, and histopathological findings. However, they were informed that the matched cohort contained an increased proportion of recurrence cases compared to typical clinical practice. Simulated ABMR protocols were designed by selecting sequences from the full-protocol MRI, segmented into 4 steps, as illustrated in Figure 1. Protocol details for each step are listed in Supplementary Table S1.

Step 1 included the most essential sequences: precontrast T1WI, early-phase T1WI, capturing cancer at its most conspicuous stage during the early arterial phase after contrast injection, and the subtracted MIP. As described by Kuhl [20], this adheres to a FAST protocol. Step 2 introduced ultrafast MIP without additional scan time beyond Step 1. Step 3 incorporated delayed-phase T1WI, requiring a waiting period after contrast injection. Step 4 added T2WI and DWI, sequences with relatively longer acquisition times. The radiologists sequentially reviewed MR images from Step 1 through Step 4. In Step 1, they documented the maximal diameter and location of any lesion categorized as BI-RADS 3, 4, or 5. Lesions were classified by type, shape, and margin as benign-looking mass, suspicious mass, or nonmass enhancement (NME). Internal enhancement patterns were assessed as homogeneous or heterogeneous, including rim enhancement. For signal intensity (SI) measurement, a circular region of interest (ROI) was manually placed on the slice where the lesion was most conspicuous and exhibited its largest diameter. For mass lesions, the ROI was drawn to encompass the majority of the lesion. For NME lesions, the ROI was positioned to include the strongly enhancing portion; while some interposed glandular tissue was inevitably included, adjacent fat or glandular tissue outside the lesion boundaries was avoided as much as possible. Typical benign lesions and background parenchymal enhancement (BPE) were not included. In Step 2, the radiologists analyzed ultrafast MIP, noting the frame numbers for aortic and lesion enhancement as well as arterial and venous drainage enhancement. The time to enhancement (TTE) and arterial–venous interval (AVI) were calculated by multiplying the frame number by the ultrafast sequence’s temporal resolution [22,23]. In Step 3, delayed-phase T1WI (approximately 7 min postcontrast injection) was reviewed, and an ROI was drawn to measure the lesion’s average SI. The enhancement pattern was categorized as washout (SI decreases by more than 10% in the delayed phase compared to the early phase), persistent (SI increases by more than 10% in the delayed phase), or plateau (SI change is less than 10% between phases). In Step 4, T2WI was used to determine whether the lesion exhibited high or Iso-to-low SI. For iso-to-low SI lesions, diffusion restriction was assessed on DWI. The average ADC value was calculated by drawing an ROI on the ADC map. Each step was reviewed at intervals of at least one week. At each step, radiologists classified cases as negative (uneventful follow-up) or positive (biopsy required). Their conclusions integrated findings from both the sequences introduced in the current step and all preceding steps.

### 2.4. Statistical Analysis

Independent two-sample *t*-tests were used to compare continuous variables, while Pearson’s chi-square tests were applied to categorical variables to analyze the clinical and pathological characteristics of the study population. Diagnostic performance for detecting recurrent breast cancer was assessed based on sensitivity, specificity, positive predictive value (PPV), negative predictive value (NPV), and overall accuracy at various stages. For each reader, the area under the curve (AUC) was calculated at each step, and Receiver Operating Characteristic (ROC) curve comparisons were conducted to evaluate differences in diagnostic performance. In addition, pairwise non-inferiority tests were performed between the steps. A non-inferiority margin of 0.05 was used, and non-inferiority was concluded if the lower bound of the 95% confidence interval (CI) for the AUC difference was greater than 0.05. Inter-reader agreement among the three radiologists was assessed using Fleiss’ kappa (κ). All statistical analyses were performed using SPSS version 25.0 (IBM) and MedCalc version 20.008 (MedCalc Software). A *p*-value < 0.05 was considered statistically significant.

## 3. Results

A total of 1002 women underwent 1002 breast MRI examinations. The baseline characteristics of the study cohort are detailed in Table 1. Before propensity score matching, the recurrence group had a higher proportion of HER2-positive cases and mastectomy patients, while the non-recurrence group included more individuals who had received radiation therapy. No significant differences were observed between the 2 groups in terms of age at diagnosis, interval between cancer diagnosis and surveillance MRI, T stage, N stage, estrogen receptor positivity, progesterone receptor positivity, neoadjuvant chemotherapy, chemotherapy, or endocrine therapy. After 1:2 propensity score matching, no significant differences remained between the recurrence and non-recurrence groups across any evaluated characteristics (Supplementary Figure S1).

**Figure 2:**
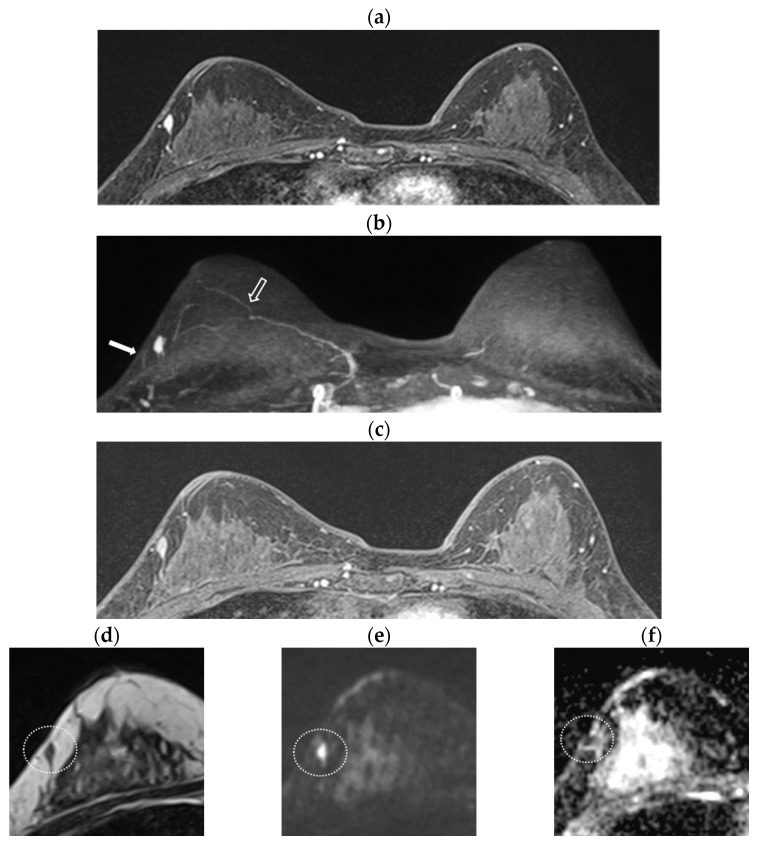
Representative MRI findings of ipsilateral breast cancer recurrence. Surveillance breast MRI of a woman who underwent breast-conserving surgery in the right upper outer quadrant three years earlier. (**a**) Early-phase T1-weighted image (T1WI) shows an oval, homogeneous enhancing mass (0.8 cm) in the right upper outer quadrant, adjacent to the prior surgical site. (**b**) Ultrafast maximal intensity projection (MIP) demonstrates rapid, intense enhancement of the lesion. The feeding artery (solid arrow) and draining vein (empty arrow) are identified; time to enhancement (TTE) was 8.4 s, and arterial–venous interval (AVI) was 16.8 s. (**c**) On delayed-phase T1WI, the lesion shows delayed washout kinetics. (**d**) The mass (dotted circle) exhibits iso-signal intensity on T2-weighted imaging (T2WI). (**e**,**f**) Diffusion-weighted images (DWIs) demonstrate diffusion restriction of the mass (dotted circle), with an apparent diffusion coefficient (ADC) value of 0.61 × 10^−3^ mm^2^/s. Pathology confirmed invasive breast cancer.

Table 2 summarizes the characteristics of the 21 recurrence cases identified in the matched cohort. Among these, 16 recurrences occurred in the ipsilateral breast (Figure 2), while 5 were detected in the contralateral breast. All 21 recurrent lesions were visible on MRI, with 20 confirmed by ultrasound-guided core needle biopsy. One lesion, presenting as grouped calcifications, was verified via stereotactic biopsy. Final pathological confirmation was obtained through surgical excision. Among the 19 lesions, 11 were invasive cancers, 2 were micro-invasive cancers, and 6 were ductal carcinoma in situ. Two lesions were metastatic carcinoma in the lymph nodes. The median MRI lesion size was 1.1 cm, ranging from 0.5 to 1.9 cm.

Table 3 presents the diagnostic performance of 4 sequential combinations of ABMR protocols across 3 readers. Sensitivity, specificity, PPV, NPV, and accuracy varied among readers. Sensitivity ranged from 76.2% to 90.5%, specificity ranged from 88.1% to 92.9%, PPV ranged from 77.3% to 85.7%, and NPV ranged from 88.6% to 92.9%. All 3 readers achieved the highest specificity at Step 4. Accuracy peaked at Step 2 for reader 1 (88.9%), at Step 4 for reader 2 (90.5%), and consistently at steps 2 through 4 for reader 3 (90.5%). No statistically significant differences were observed in the AUC between steps for each reader (*p* > 0.05). The diagnostic performance of successive ABMR protocol steps was evaluated using non-inferiority testing based on AUC differences across three radiologists. All pairwise comparisons between ABMR protocol steps demonstrated non-inferiority, as the lower bounds of the 95% CIs in all cases were above the predefined margin of –0.05. Inter-reader agreement, assessed using Fleiss’ kappa, indicated substantial agreement at each step, with κ values of 0.601 at Step 1, 0.674 at Step 2, 0.648 at Step 3, and 0.661 at Step 4.

Table 4 compares the MRI characteristics of malignant and benign breast lesions. Among the parameters evaluated, delayed-phase T1 SI was significantly lower in malignant lesions compared to benign lesions (220 vs. 249, *p* = 0.027), with a significant difference in the distribution of delayed kinetic patterns (washout, plateau, and persistent) between the 2 groups (*p* < 0.001). Similarly, the ADC value was significantly lower in malignant lesions than in benign ones (0.77 vs. 1.04, *p* = 0.037). However, ADC values could only be measured in 43% (21/49) of lesions, highlighting a limitation in evaluating diffusion characteristics across all cases. Other parameters, including lesion type, internal enhancement, early-phase T1WI SI, TTE, AVI, and T2WI SI, showed no statistically significant differences between malignant and benign lesions.

## 4. Discussion

This study aimed to determine the optimal ABMR protocol for PHBC surveillance. Simulated ABMR protocols demonstrated consistent diagnostic performance for recurrence detection, with no statistically significant differences. Delayed-phase post-contrast imaging and DWI aided in differentiating benign from malignant lesions, though diagnostic performance metrics showed no significant variation.

Among readers, sensitivity ranged from 76.2% to 90.5% and specificity ranged from 88.1% to 92.9%, indicating reliable performance across protocol steps. However, ABMR sensitivity and specificity in this study were slightly lower than those in previous reports (80–100% and 82.2–95.3%, respectively) [14,15,16,17,18,19]. This variation may be due to differences in the establishment of reference standards. Previous research primarily compared ABMR with full-protocol MRI or other imaging modalities, such as mammography and ultrasound. In contrast, this study employed a comprehensive reference standard integrating findings from all imaging modalities, including mammography, ultrasound, and full-protocol MRI, while also incorporating comparisons with prior imaging. Similarly, Kwon et al. [24] reported reduced sensitivity (71.4%) when evaluating ABMR alone, using a reference standard derived from longitudinal medical records of imaging surveillance and biopsy results, aligning with our findings. Additionally, this study focused on identifying the optimal sequence combination rather than assessing ABMR’s overall diagnostic performance. A matched cohort with a higher recurrence proportion than typically observed in clinical practice was created, which may have influenced diagnostic performance outcomes.

Step 1 (FAST protocol) demonstrated reliable diagnostic performance, with sensitivity at 81%, specificity ranging from 88.1% to 90.5%, PPV ranging from 77.3% to 81%, NPV ranging from 90.2% to 90.5%, and accuracy ranging from 85.7% to 87.3%. Although overall performance was slightly lower than in Step 4, the differences were not statistically significant, indicating that the addition of subsequent sequences did not provide a significant incremental diagnostic advantage over Step 1 in this study population. These results are consistent with the findings of a study comparing ABMR and full protocol MRI for screening in women with extremely dense breasts [25]. In that study, an abbreviated protocol—including both high-temporal low-spatial and low-temporal high-spatial dynamic T1-weighted series up to 120 s after contrast agent injection—maintained high diagnostic accuracy, while reducing reading and scanning times.

The addition of ultrafast MIP sequences resulted in a slight AUC increase across all readers, though these differences were not statistically significant. All readers showed a modest NPV and accuracy increase, with reader 1 also exhibiting a slight improvement in specificity. These findings align with previous studies [26,27], which reported that incorporating ultrafast MIP sequences into ABMR enhanced specificity and PPV, reducing unnecessary short-term follow-ups. To improve clinical applicability, this study used TTE and AVI, assessed via frame numbers from ultrafast MIP sequences, instead of constructing kinetic curves from ultrafast DCE-MRI. TTE and AVI assessments, which did not require kinetic curve plotting, were shorter in malignant lesions, consistent with previous reports [22,28,29]; however, the differences were not statistically significant, likely due to the limited number of evaluated lesions. The small lesion size presented challenges, as CS reconstruction, despite its high spatial resolution, sometimes rendered lesion evaluation unfeasible, particularly when assessing arterial and venous structures. One advantage of ultrafast sequences is their ability to minimize BPE effects, improving lesion distinction [28,30,31]. However, in this study, 75% of women had minimal BPE, and 16% had mild BPE, suggesting that most patients exhibited low BPE levels, likely due to postoperative adjuvant therapy. This may have reduced the ultrafast sequence’s practical benefit.

Delayed-phase T1 imaging is well recognized for its role in lesion characterization through kinetic curve analysis. In this study, it was successfully evaluated in all breast lesions and demonstrated significant parameter differences between malignant and benign lesions. However, its influence on reader decisions was minimal, as most were made in earlier steps, with the delayed phase primarily used to confirm rather than modify assessments. This finding aligns with Kim et al. [15], who reported that delayed-phase MRI has a limited role and that ABMR offers comparable sensitivity to full-protocol MRI, supporting reduced reliance on delayed-phase imaging in ABMR.

In Step 4, reader 1’s performance remained unchanged, while Readers 2 and 3 showed marginal specificity and PPV improvements. These variations were more likely driven by DWI sequences rather than T2WI, as few lesions exhibited high SI on T2WI, and the exclusion of BI-RADS category 2 benign lesions limited their evaluative relevance. Similarly, Heacock et al. [32] reported that incorporating a T2-weighted sequence did not significantly alter the cancer detection rate. Conversely, ADC values provided clear malignant–benign contrasts, facilitating differentiation. However, ADC measurements were feasible in 43% (21/49) of lesions, primarily due to small lesion size. Notably, one-third (7/21) of the recurrence cases were subcentimeter lesions. Given the relatively poor image resolution and thicker slices of DWI compared to T1WI, accurate measurement of such small lesions was often limited. Nonetheless, this limitation should not be interpreted as evidence against the effectiveness of including DWI sequences in ABMR protocols. Despite the challenges of evaluating small lesions, ADC values showed a significant difference between malignant and benign lesions (*p*-value = 0.037), and two readers showed improved specificity and PPV in the final step. These findings suggest that DWI provides valuable complementary information for lesion characterization.

This study has several limitations. First, it was a retrospective study conducted at a single tertiary care center, primarily involving Asian women. All imaging was performed using 3T MRI scanners within a national health insurance system that provides reimbursement for breast MRI. These factors may limit the generalizability of our findings in other clinical environments, particularly those with more diverse populations, greater reliance on 1.5T MR scanners, and different healthcare systems. Second, although the source cohort was large, the final matched cohort included a small number of recurrence cases, which limited the statistical power and reduced the precision of diagnostic metrics. As a result, subtle differences between protocols may not have been detectable. While primary tumor characteristics and treatment factors were matched between the recurrence and non-recurrence groups, MRI findings, including suspicious lesions and subsequent biopsy outcomes, were not considered in the matching process, which may have introduced confounding effects. The abbreviated protocols were simulated by extracting sequences from the full protocol and interpreted in separate sessions. While this approach allows for controlled comparison of different sequence combinations, it does not replicate the operational characteristics of real-world ABMR acquisition, particularly in terms of reduced scan time, improved patient comfort, and increased susceptibility to motion artifacts. Despite a large surveillance cohort, not all eligible patients were included, potentially introducing selection bias. Lastly, the follow-up for negative exams was limited to at least 12 months, which may have been insufficient for confirmation of a lesion’s long-term stability or benign/non-cancerous nature.

Therefore, prospective validation using dedicated ABMR acquisitions is needed to assess the real-world applicability of these findings. In particular, larger multicenter studies involving diverse populations are necessary to confirm our results and determine their broader relevance in clinical practice. In addition, comparisons with alternative imaging modalities—especially contrast-enhanced mammography, which is gaining attention as a promising option in low-resource settings—may also help define optimal strategies across diverse healthcare environments [33].

In conclusion, this study demonstrated comparable diagnostic performances across ABMR protocols. The FAST sequence alone proved reliable, with additional sequences providing only modest improvements.

## Figures and Tables

**Table 1:** Baseline characteristics of the study cohort.

	Unmatched Cohort			Propensity Score-Matched Cohort		
Characteristics	No Recurrence (*n* = 979)	Recurrence (*n* = 23)	*p* Value	No Recurrence (*n* = 42)	Recurrence (*n* = 21)	*p* Value
Age at diagnosis (years)	49.7 ± 9.9	47.5 ± 7.5	0.089	48.9 ± 8.8	47.8 ± 7.6	0.636
Interval (months)	24 [12, 45.3]	44 [14, 51]	0.068	36 [11.8, 52.5]	43.5 [15, 50.5]	0.436
T stage			0.433			0.819
T0	205 (21)	8 (35)		18 (43)	8 (38)	
T1	519 (52)	8 (35)		15 (36)	7 (33)	
T2	245 (25)	7 (30)		9 (21)	6 (29)	
T3	10 (1)	0		0	0	
N stage			0.559			0.844
N0	813 (83)	17 (74)		35 (83)	17 (81)	
N1	127 (13)	4 (17)		5 (12)	3 (14)	
N2	22 (2)	1 (4)		1 (2)	1 (5)	
N3	17 (2)	1 (4)		1 (3)	0	
Estrogen receptor positive	822 (84)	19 (83)	0.814	28 (67)	17 (81)	0.375
Progesterone receptor positive	764 (78)	16 (70)	0.355	25 (60)	14 (67)	0.783
HER2 positive	196 (20)	9 (39)	0.022	14 (43)	9 (33)	0.358
Type of surgery			0.018			0.923
Breast conserving therapy	813 (83)	14 (61)		30 (71)	14 (67)	
Mastectomy	166 (17)	9 (39)		12 (29)	7 (33)	
Type of therapy						
Neoadjuvant chemotherapy	78 (8)	2 (9)	0.705	19 (45)	7	0.702
Radiation therapy	881 (90)	15 (65)	0.007	29 (69)	14	1
Chemotherapy	352 (36)	8 (35)	0.912	13 (31)	7	1
Endocrine therapy	842 (86)	19 (83)	0.688	28 (67)	17	0.375

HER2, Human epidermal growth factor receptor 2.

**Table 2:** Lesion characteristics of recurrence cases.

No	Location	MRI Size (cm)	Recurrence Pathology
1	contralateral breast	1.1	DCIS, high grade
2	contralateral breast	1.2	invasive cancer, grade 2
3	ipsilateral breast	0.5	DCIS, intermediate grade
4	ipsilateral breast	1.3	invasive cancer, grade 2
5	ipsilateral breast	1.2	invasive cancer, grade 2
6	ipsilateral axillary lymph node	1.2	metastatic carcinoma
7	contralateral breast	1.3	invasive cancer, grade 2
8	contralateral breast	5	micro-invasive cancer
9	ipsilateral breast	0.5	DCIS, low grade
10	contralateral breast	1.1	micro-invasive cancer
11	ipsilateral internal mammary lymph node	1.4	metastatic carcinoma
12	ipsilateral breast	0.6	invasive cancer, grade 2
13	ipsilateral nipple	0.8	DCIS, intermediate grade (Paget’s disease)
14	ipsilateral chest wall	1	invasive cancer, grade 2
15	ipsilateral breast	0.9	invasive cancer, grade 1
16	ipsilateral breast	1.3	invasive cancer, grade 2
17	ipsilateral chest wall	0.5	invasive cancer, grade 2
18	ipsilateral chest wall	0.6	invasive cancer, grade 3
19	ipsilateral breast	1.5	DCIS, intermediate grade
20	ipsilateral chest wall	1.9	invasive cancer, grade 2
21	contralateral breast	2	DCIS, intermediate grade

DCIS, ductal carcinoma in situ.

**Table 3:** Diagnostic performances of different combinations of abbreviated breast MRI.

	Reader 1				Reader 2				Reader 3			
	Step 1	Step 2	Step 3	Step 4	Step 1	Step 2	Step 3	Step 4	Step 1	Step 2	Step 3	Step 4
Sensitivity (%)	81.0	81.0	76.2	76.2	81.0	85.7	85.7	85.7	81.0	90.5	90.5	85.7
Specificity (%)	88.1	92.9	92.9	92.9	88.1	88.1	88.1	92.9	90.5	90.5	90.5	92.9
PPV (%)	77.3	85	84.2	84.2	77.3	78.3	78.3	85.7	81.0	82.6	82.6	85.7
NPV (%)	90.2	90.7	88.6	88.6	90.2	92.5	92.5	92.9	90.5	95	95	92.9
Accuracy (%)	85.7	88.9	87.3	87.3	85.7	87.3	87.3	90.5	87.3	90.5	90.5	90.5
AUC	0.83	0.87	0.85	0.85	0.83	0.87	0.87	0.89	0.83	0.91	0.91	0.89

PPV, positive predictive value; NPV, negative predictive value; AUC, area under the curve.

**Table 4:** MRI characteristics of detected malignant and benign breast lesions.

Parameters (*n* = 49)	Malignant (*n* = 19)	Benign (*n* = 30)	*p*-Value
early-phase T1 lesion type			0.363
benign appearing mass	5 (26)	14 (46)	
suspicious mass	7 (37)	8 (27)	
nonmass enhancement	7 (37)	8 (27)	
early-phase T1 internal enhancement			0.271
homogeneous	9 (47)	19 (63)	
heterogeneous	10 (53)	11 (37)	
early-phase T1 SI	237 [212, 265]	242 [201, 159]	0.116
ultrafast TTE (*n* = 39)	12.6 [8.4, 18.9]	16.8 [12.6, 21]	0.427
ultrafast AVI (*n* = 31)	16.8 [16.8, 21]	21 [16.8, 29.4]	0.681
delayed-phase T1 SI	220 [185, 257]	262 [223, 326]	0.002
delayed-phase kinetics			<0.001
delayed washout	8 (42)	1 (3)	
delayed plateau	7 (37)	9 (30)	
delayed persistent	4 (21)	20 (67)	
T2WI high SI (*n* = 48)			0.241
high SI	0	2 (7)	
iso-to-low SI	19 (100)	28 (93)	
ADC value (×10^−3^ mm^2^/s) (*n* = 21)	0.77 [0.63, 0.97]	1.04 [0.90, 1.66]	0.037

Data are presented as numbers (percentage) and medians [interquartile range]; TTE, time to enhancement; AVI, arterial venous interval; ADC, apparent diffusion coefficient.

## Data Availability

All data generated and analyzed during this study are included in this published article. Raw data supporting the findings of this study are available from the corresponding author on request.

## Supplementary Materials

The following supporting information can be downloaded at https://www.mdpi.com/article/10.3390/diagnostics16081138/s1. Figure S1: Covariate balance before and after matching; Table S1: Summary of imaging sequences for each AB-MRI protocol step.

## Abbreviations

The following abbreviations are used in this manuscript:

ABMR	Abbreviated breast MRI
AUC	Area under the curve
ADC	Apparent diffusion coefficient
AVI	Arterial–venous interval
BPE	Background parenchymal enhancement
BI-RADS	Breast Imaging Reporting and Data System
DCE-MRI	Dynamic contrast-enhanced MRI
DWI	Diffusion-weighted imaging
HER2	Human epidermal growth factor receptor 2
MIP	Maximum intensity projection
MRI	Magnetic resonance imaging
NPV	Negative predictive value
PHBC	Personal history of breast cancer
PPV	Positive predictive value
ROC	Receiver operating characteristic
SI	Signal intensity
TTE	Time to enhancement
