# Supplementary material for: Optimizing Abbreviated Breast MRI for Surveillance in Women with Personal History of Breast Cancer
Source: Diagnostics (Basel). 2026 Apr 10;16(8):1138. doi: 10.3390/diagnostics16081138 (PMC13114410; doi:10.3390/diagnostics16081138)

**Supplementary Figure S1.** Covariate balance before and after matching.

Standardized mean differences of key covariates between groups are plotted to assess balance before (Unmatched, orange) and after (Matched, green) propensity score matching. Improved balance post-matching suggests reduced baseline differences.

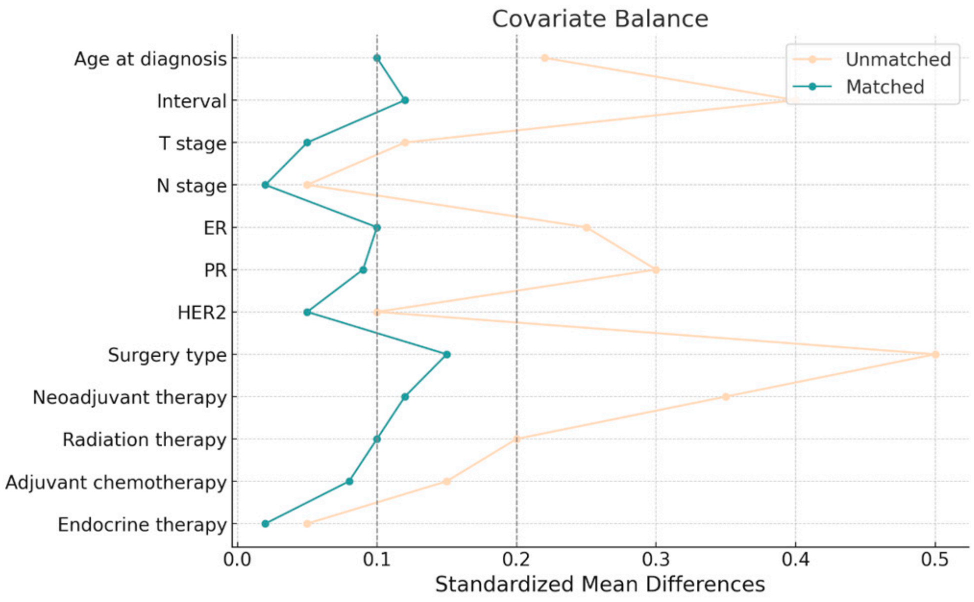

Supplement: Supplementary file 1 [file diagnostics-16-01138-s001.zip › Supplementary Figure S1.pdf]
