# Supplementary material for: Optimizing Abbreviated Breast MRI for Surveillance in Women with Personal History of Breast Cancer
Source: Diagnostics (Basel). 2026 Apr 10;16(8):1138. doi: 10.3390/diagnostics16081138 (PMC13114410; doi:10.3390/diagnostics16081138)
Supplement: Supplementary file 1 [file diagnostics-16-01138-s001.zip › Supplementary Table S1.pdf]

Supplementary Table S1. Summary of Imaging Sequences for Each AB-MRI Protocol Step.

Step	Included Sequences	Key Imaging Parameters
Step 1	Precontrast T1WI<br>Early-phase T1WI & Subtracted MIP; 93 sec after contrast injection	3D T1-weighted FLASH<br>TR/TE 4.7/2.3 ms<br>Matrix: 448×358; Slice thickness 1.0 mm
Step 2	Ultrafast DCE MIP; starts with contrast injection; lasts 84 sec	3D FS VIBE with CS<br>TR/TE 3.1/1.2 ms<br>Matrix: 352×352; Slice thickness 1.0 mm<br>Temporal resolution 4.2 sec
Step 3	Delayed-phase T1WI; 425 sec after contrast injection	3D T1-weighted FLASH<br>TR/TE 4.7/2.3 ms<br>Matrix: 448×358; Slice thickness 1.0 mm
Step 4	T2WI<br>DWI (b = 0, 1000 s/mm²)<br>ADC map	TR/TE 5000/96 ms; Slice thickness 3 mm<br>TR/TE 4720/60 ms; Slice thickness 3 mm

T1WI = T1-weighted imaging; MIP = maximum intensity projection; FLASH = fast low-angle shot; TR = repetition time; TE = echo time; DCE = dynamic contrast-enhanced; FS = fat-suppressed; VIBE = volumetric interpolated breath-hold examination; CS = compressed sensing; T2WI = T2-weighted imaging; DWI = diffusion-weighted imaging; ADC = apparent diffusion coefficient
